# Solitary Fibrous Tumor of the Oropharyngeal Lateral Wall: A Case Report and Review of Diagnostic and Therapeutic Challenges

**DOI:** 10.7759/cureus.100400

**Published:** 2025-12-30

**Authors:** Muhammad Zahlaka, Abdellatif Zhalka, Daniel Gabis, Basel Jabarin, Abraham Goldfarb, Shadi Shinnawi

**Affiliations:** 1 Otolaryngology - Head and Neck Surgery, Wolfson Medical Center, Holon, ISR; 2 Endocrinology, Emek Medical Center, Kafr Qara, ISR; 3 Otolaryngology - Head and Neck Surgery, University of California Davis Health System, Sacramento, USA

**Keywords:** head and neck tumor, nab2-stat6, oropharynx cancer, recurrence, solitary fibrous tumor

## Abstract

Solitary fibrous tumors (SFTs) of the head and neck are rare, and involvement of the oropharynx is very uncommon. We present the case of an 85-year-old woman who developed a rapidly growing SFT from the lateral pharyngeal wall. This growth caused significant airway blockage and required urgent surgical removal. After the initial surgery, the tumor recurred quickly and resulted in considerable health problems. Histopathology showed typical SFT characteristics, and immunohistochemistry confirmed the diagnosis with strong STAT6 nuclear staining. Risk assessment placed the patient in the intermediate-risk category for metastasis and tumor-related death. The patient chose not to pursue further cancer treatment. Early recurrence was noted, and she later passed away from unrelated causes.

This report highlights the challenges in diagnosing and treating oropharyngeal SFTs. It emphasizes the need for molecular confirmation and adds to the limited research on SFTs in rare head and neck locations.

## Introduction

Solitary fibrous tumors (SFTs) are rare mesenchymal tumors first identified in the pleura [1], but they are now found in almost every part of the body, including the central nervous system, abdominal cavity, retroperitoneum, soft tissues, and, notably, the head and neck region. In the past, related conditions such as hemangiopericytoma (HPC), giant cell angiofibroma (GCA), and orbital fibrous histiocytoma (OFH) were thought to be separate diagnoses [2]. However, recent developments in molecular pathology, particularly the discovery of the recurring NAB2-STAT6 gene fusion and the use of STAT6 immunohistochemistry [3], have brought these entities together under the broader SFT category, according to the current WHO classification [4].

Head and neck SFTs are exceptionally rare, making up only 6%-18% of all SFTs and about a quarter of extrathoracic cases. In the head and neck region, the most common locations are the sinonasal tract, orbit, and oral cavity, while occurrences in the oropharynx are extremely rare [1,5-7]. To date, fewer than 10 well-documented cases of primary SFT/HPC in the oropharynx have been reported in the literature, with those arising from the lateral pharyngeal wall being particularly unusual and, to our knowledge, not previously described in detail.

These tumors exhibit significant histological variability and typically show a “patternless pattern” of spindle cells and prominent staghorn vasculature [8]. They can closely resemble other soft tissue or vascular tumors, making diagnosis challenging, especially in uncommon locations. The key immunohistochemical feature is nuclear STAT6 positivity, which is highly sensitive and specific for diagnosing SFT in conjunction with characteristic histology and clinical features.

The clinical behavior of SFTs is unpredictable. Most SFTs tend to have a benign course; however, local recurrence and distant metastasis can occur, even many years after the initial diagnosis. Risk stratification models, such as the one proposed by Demicco et al. [9], consider patient age, tumor size, mitotic activity, and necrosis when predicting outcomes. However, their use in head and neck cases is debated due to limited data. SFTs in the head and neck often show a higher likelihood of local recurrence rather than metastasis, especially when surgical removal is incomplete [2,9].

The rare presentation, varied clinical course, and diagnostic challenges of head and neck SFTs, especially in the oropharynx, provide valuable opportunities to deepen our understanding of this condition. In this report, we detail the case of a patient who developed a rapidly growing oropharyngeal SFT, emphasizing the importance of molecular diagnostics and the distinct management challenges in older populations.

## Case presentation

An 85-year-old woman, without any major medical issues in her past, went to the Emergency Department after experiencing increasing nasal obstruction, a reduced ability to eat, and trouble using her dentures for about three months. A physical exam revealed a large submucosal mass that was almost completely blocking the oropharyngeal airway, though the exact origin was unclear. A nasal fiberoptic endoscopy showed a bulky submucosal tumor, leading to complete blockage of both nasal choanae.

**Figure 1:**
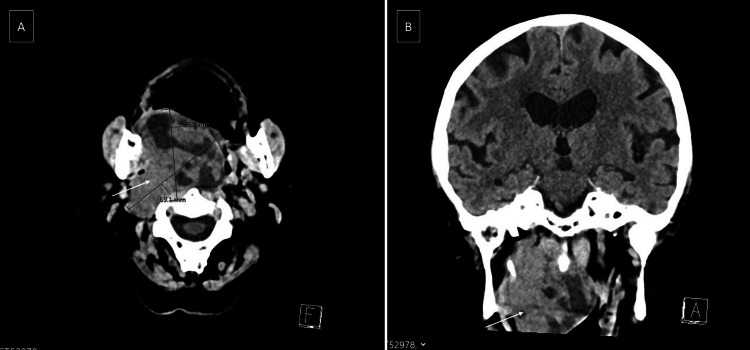
A) Axial CT showing a large, heterogeneous mass filling the entire oropharyngeal lumen (white arrow); B) same finding on coronal CT (white arrow).

A contrast-enhanced CT scan of the head and neck revealed a large, uneven mass with areas of lower density, filling both the nasopharynx and oropharynx, again with an uncertain origin. Axial and coronal images confirmed an extensive lesion almost entirely occupying the oropharyngeal space (Figure 1).

Biopsies taken from both the oropharynx and nasopharynx in the clinic were non-diagnostic. Due to uncertainty in diagnosis and serious airway issues, the patient was scheduled for an operative biopsy and removal of part of the mass. During the surgery, a submucosal tumor was seen, starting just behind the right palatine tonsil and extending upward toward the soft palate and nasopharynx. Some areas of dead tissue were found, but there was no sign of excessive bleeding during the operation.

**Figure 2:**
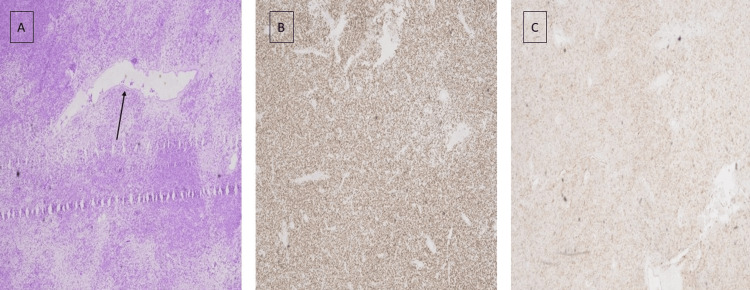
Histological examination: A) H&E stain showing a staghorn configuration (black arrow); B) positive STAT6 stain; C) positive CD34 stain.

Histopathological examination showed a growth of spindle-shaped cells with a disorganized structure and distinctive staghorn-shaped blood vessels. Immunohistochemistry showed strong nuclear STAT6 positivity and some CD34 expression, confirming the diagnosis of an SFT, previously known as HPC (Figure 2).

A risk assessment for the chance of metastasis and tumor-related death was carried out based on the four-parameter Demicco et al. model [10]. This model includes patient age, tumor size, mitotic count, and the extent of necrosis in the tumor. In this case, the parameters were: the patient’s age was 85, the tumor measured 7 cm, there were 6 mitoses per 10 high-power fields, and at least 10% of the tumor showed necrosis. These results corresponded to a total score of 5 in the Demicco model, placing the patient in the intermediate-risk category for metastasis and tumor-related death.

The patient chose not to pursue further cancer treatment. During follow-up one month after surgery, the tumor had rapidly regrown to its original pre-surgery size, but the patient still did not experience any airway issues. Six months after the initial diagnosis, the patient passed away from an unrelated cause - an intracranial hemorrhage due to head trauma.

## Discussion

SFTs present a clinical and diagnostic challenge due to their diverse histologic features and unpredictable behavior [10]. Oropharyngeal involvement is rare, particularly when the tumor arises from the lateral wall. This complicates early diagnosis and the creation of standardized treatment plans. Epidemiological data show that SFTs do not favor one gender and usually present at a median age of 52 years, with a mean tumor diameter of 2.8 cm². In this case, the patient’s advanced age and the unusually large oropharyngeal mass highlight the variability in clinical presentation and the possibility of atypical cases in this rare disease.

The shift from the old classification of HPC to the unified SFT spectrum has been driven by progress in molecular genetics. The detection of NAB2-STAT6 fusion and STAT6 immunostaining has become an important diagnostic tool in recent years [3,11]. CD34 is still a significant marker, though not fully specific [8], and interpreting it alongside the morphology is crucial [12].

Our case illustrates important issues in managing SFTs. The patient’s age and significant tumor size show that SFTs can present aggressively, even in older adults [10]. Despite comprehensive radiologic examinations, preoperative imaging could not pinpoint the tumor's exact origin, highlighting the limits of imaging in large head and neck masses, and the importance of thorough intraoperative assessment [13]. The risk assessment for our patient, using the Demicco et al. model - considering age, tumor size, mitoses, and necrosis - placed her in the intermediate-risk category for metastasis and tumor-related death. While this model is commonly used, its effectiveness for head and neck SFTs is uncertain. These tumors often have a higher local recurrence rate, but a lower chance of spreading compared to similar tumors in other areas [2,12]. Early recurrence, as in this case, is well recognized, especially when surgical removal is incomplete or only palliative debulking is performed [2,14]. This raises questions about the best surgical approach and follow-up, particularly for patients who cannot or do not wish to undergo additional definitive treatment.

Surgical resection is still the main treatment option. However, radiotherapy and chemotherapy mainly play supportive roles and are reserved for cases that cannot be surgically removed, are recurrent, or have malignant features [9,14]. Molecular diagnostics, such as confirming the NAB2-STAT6 fusion, are now essential for diagnosis and may help with targeted treatment in the future [3,11,12]. As more cases are documented, shared experience will continue to improve prognostic tools, treatment plans, and monitoring strategies for this rare condition.

## Conclusions

SFTs of the head and neck, particularly those arising in the oropharynx, remain exceptionally rare and diagnostically demanding. This case illustrates the necessity of molecular diagnostics for histologic confirmation, the challenge of preoperative anatomic mapping in large tumors, and the limitations of current risk models for predicting clinical outcomes in head and neck SFTs. Recognition of their local aggressiveness, especially in elderly patients unable to undergo radical surgery, emphasizes the importance of a comprehensive workup, individualized management plans, and vigilant long-term surveillance. The accumulation of further case reports will be crucial for refining prognostic stratification and improving patient care for this uncommon neoplasm.
